# Surveys in northern Utah for egg parasitoids of *Halyomorphahalys* (Stål) (Hemiptera: Pentatomidae) detect *Trissolcusjaponicus* (Ashmead) (Hymenoptera: Scelionidae)

**DOI:** 10.3897/BDJ.8.e53363

**Published:** 2020-08-13

**Authors:** Mark Cody Holthouse, Zachary R Schumm, Elijah J Talamas, Lori R Spears, Diane G Alston

**Affiliations:** 1 Department of Biology, Utah State University, Logan, United States of America Department of Biology, Utah State University Logan United States of America; 2 Florida State Collection of Arthropods, Gainesville, FL, United States of America Florida State Collection of Arthropods Gainesville, FL United States of America; 3 Systematic Entomology Laboratory, Washington, DC, United States of America Systematic Entomology Laboratory Washington, DC United States of America

**Keywords:** parasitoid wasp, stink bug, egg mass, biological control

## Abstract

The highly polyphagous and invasive brown marmorated stink bug, *Halyomorphahalys* (Stål) (Hemiptera: Pentatomidae), has become a significant insect pest in North America since its detection in 1996. It was first documented in northern Utah in 2012 and reports of urban nuisance problems and plant damage have since increased. Biological control is the preferred solution to managing *H.halys* in North America and other invaded regions due to its alignment with integrated pest management and sustainable practices. Native and non-native biological control agents, namely parasitoid wasps, have been assessed for efficacy. *Trissolcusjaponicus* (Ashmead) (Hymenoptera: Scelionidae) is an effective egg parasitoid of *H.halys* in its native range of southeast Asia and has recently been documented parasitising *H.halys* eggs in North America and Europe. Field surveys for native and exotic egg parasitoids using wild (*in situ*) and lab-reared *H.halys* egg masses were conducted in suburban and agricultural sites in northern Utah from June to September 2017–2019. Seven native wasp species in the families Eupelmidae and Scelionidae were discovered guarding *H.halys* eggs and adult wasps from five of these species completed emergence. Native species had low mean rates of adult emergence from wild (0.5–3.7%) and lab-reared (0–0.4%) egg masses. In 2019, an adventive population of *T.japonicus* was discovered for the first time in Utah, emerging from 21 of the 106 wild *H.halys* egg masses found that year, and none from lab-reared eggs. All *T.japonicus* emerged from egg masses collected on *Catalpaspeciosa* (Warder). Our results support other studies that have observed biological control of *H.halys* from *T.japonicus* and improved parasitoid wasp detection with wild as compared to lab-reared *H.halys* egg masses.

## Introduction

The brown marmorated stink bug, *Halyomorphahalys* (Stål) (Hemiptera: Pentatomidae), is a severe agricultural and urban nuisance pest that originates from southeast Asia (Hoebeke and Carter 2003) and has invaded numerous countries worldwide (Cesari et al. 2015, Gariepy et al. 2014, Haye et al. 2015b, Macavei et al. 2015, Milonas and Partsinevelos 2014, Vétek et al. 2014). As of 2020, it has been detected in 46 U.S. States and four Canadian Provinces, with 11 States reporting severe agricultural damage (StopBMSB.org). *Halyomorphahalys* was first detected in Utah in 2012 and has been considered a pest to fruit and vegetable crops since 2017. With the threat of increasing economic agricultural damage, development of proactive management tactics is imperative. In the U.S. Mid-Atlantic region, where *H.halys* has been a severe pest, effective control has relied on broad spectrum insecticides, leading to increased application frequency and disruption of integrated pest managment, including secondary pest outbreak (Lee et al. 2014, Leskey et al. 2014, Leskey et al. 2012a, Leskey et al. 2012b). Physical or cultural control (e.g. trap cropping and mass trapping) can offer some mitigation of plant damage, but may not be economically viable (Mathews et al. 2017). The most effective management tactics have paired cultural and chemical tactics (e.g. orchard perimeter insecticide applications and treatment of trap trees) (Blaauw et al. 2014).

Biological control by egg parasitoids has proven effective in suppressing *H.halys* populations in its native range (Yang et al. 2009, Zhang et al. 2017). *Halyomorphahalys* sentinel egg mass surveys in North America have identified parasitism by native parasitoids in the families Scelionidae, Encyrtidae and Eupelmidae (Abram et al. 2017, Cornelius et al. 2016a, Cornelius et al. 2016b, Balusu et al. 2019, Talamas et al. 2015a, Talamas et al. 2015b). However, parasitism rates are low, likely due to inability of native species to overcome healthy *H.halys* egg defences (Abram et al. 2017, Abram et al. 2014, Dieckhoff et al. 2017,Herlihy et al. 2016). Measuring native parasitoid effectiveness against *H.halys* eggs solely by wasp emergence may underestimate their impact, as partial development of a native wasp inside *H.halys* eggs can cause egg mortality (Abram et al. 2019a, Abram et al. 2014, Cornelius et al. 2016a, Schumm 2020). Therefore, evaluating native wasp parasitism rates, especially in novel landscapes where new behaviour or species may be observed, deserves critical analysis.

*Trissolcusjaponicus* (Ashmead) (Hymenoptera: Scelionidae) is an egg parasitoid native to the home range of *H.halys* (Yang et al. 2009). Adventive *T.japonicus* have been discovered emerging from *H.halys* egg masses in North America (Milnes et al. 2016, Talamas et al. 2015b) and Europe (Stahl et al. 2019, Sabbatini Peverieri et al. 2018). Research has assessed the effectiveness of these adventive populations against *H.halys* and a recent study in Washington State revealed parasitoid emergence rates reaching 77% (Milnes and Beers 2019). Conversely, initial parasitism of *H.halys* eggs by *T.japonicus* in Europe has been as low as 2%. (Stahl et al. 2019).

Though adult wasp emergence has been documented on eggs of some native pentatomidae species, *Trissolcusjaponicus* has shown superior adult emergence rates on *H.halys* eggs (Milnes and Beers 2019). Laboratory paired-host tests demonstrated significantly higher *T.japonicus* parasitism rates of *H.halys* over other stink bug species. However, no-choice tests documented *T.japonicus* readily parasitising *Banasadimidiata* (Say) and *Holcostethusabbreviatus* Uhler (Hedstrom et al. 2017). Recent field tests in the Pacific Northwest found significantly lower *T.japonicus* parasitism rates of native stink bug egg masses (0.4–⁠8%) compared to *H.halys* (77%) (Milnes and Beers 2019). These findings suggest that, although non-target effects occur, natural settings may support more targeted control of *H.halys* by *T.japonicus*.

The primary objective of this study was to utilise *H.halys* egg mass surveys to identify potential parasitoid species for suppression of this invasive insect pest in northern Utah. Northern Utah provides novel geographic and environmental conditions for detection of H.halys parasitoids, most notably high elevation (>1200 m) and arid sites with a hot summer and cold winter climate (Utah Climate Center 2020), as compared to other regions where *H.halys* occurs (Herlihy et al. 2016, Jarrett et al. 2019, Milnes et al. 2016, Stahl et al. 2019). Secondly, we compared parasitism rates of wild (*in situ*) versus lab-reared egg masses to better understand effective survey approaches and projection of natural parasitism rates in the field (Abram et al. 2017).

## Materials and Methods

### Survey Sites

**Figure 1.**
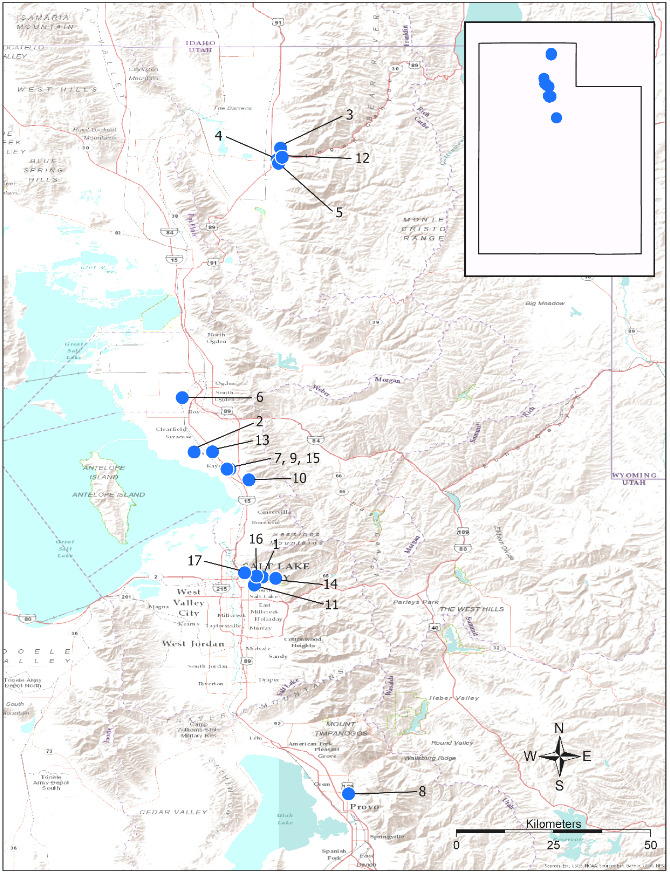
Blue dots indicate deployment and collection sites of lab-reared and wild egg masses in northern Utah, 2017-2019. *Trissolcusjaponicus* was discovered at Sites 1 and 17. Geographical coordinates are as follows: Site 1: 40°46'14.8"N, 111°51'18.6"W; Site 2: 41°3'36.899"N, 112°0'46.944"W; Site 3: 41°45'48.39"N, 111°48'46.148"W; Site 4: 41°44'11.685"N, 111°49'14.446"W; Site 5: 41°43'41.968"N, 111°49'4.005"W; Site 6: 41°11'9.164"N, 112°2'25.368"W; Site 7: 41°1'18.787"N, 111°55'59.663"W; Site 8 40°16'07.6"N, 111°39'20.7"W; Site 9: 41°01'12.2"N, 111°55'49.4"W; Site 10: 40°59'44.2"N, 111°53'09.8"W; Site 11: 40°45'08.2"N, 111°52'25.2"W; Site 12: 41°44'32.9"N, 111°48'32.8"W; Site 13: 41°03'37.0"N, 111°58'13.8"W; Site 14: 40°46'03.3"N, 111°49'27.5"W; Site 15: 41°01'13.1"N, 111°56'12.9"W; Site 16: 40°46'23.0"N, 111°52'07.3"W; and Site 17: 40°46'49.3"N, 111°53'46.5"W.

Surveys for native and exotic parasitoid wasps of *H.halys* eggs in northern Utah were conducted from June through September in each of 2017, 2018 and 2019. The surveys included a total of 17 field sites. Sites 1, 4, 5, 8 and 10–⁠17 were located in suburban landscapes containing mixed woody ornamental trees and shrubs. Sites 2, 3, 6, 7 and 9 were in conventionally-managed agricultural row crops and orchards (Fig. 1). Survey sites were chosen, based on areas of established *H.halys* populations and preferred host plant availability (Tables 1, 2).

### Stink Bug Colony

*Halyomoprhahalys* egg masses were reared in the Department of Biology at Utah State University, Logan, Utah. The colony was initiated and continuously supplemented from wild *H.halys* collections in northern Utah beginning in 2016 and further supplemented in 2019 by egg masses from a colony at the New Jersey Department of Agriculture in Trenton, New Jersey. The lab colony was maintained at 25–⁠28°C, 40–⁠60% RH, with a 16:8 hr photoperiod.

### Survey Methods

Fresh lab-reared egg masses were deployed at field sites within 24–⁠48 hr post-oviposition. All lab-reared egg masses were oviposited on to paper towels, assessed for the number of eggs they contained and attached to wax-covered cardstock (4 cm x 4 cm), using double-sided sticky tape with sand to cover excess adhesive before field deployment. Lab-reared egg masses mounted on cardstock were attached to the underside of plant leaves (Table 1) 2–⁠3 m above the ground using metal safety pins and collected approximately 48 hr after deployment The number of lab-reared egg masses deployed each season was dependent on the lab colony fecundity: 114, 93 and 28 in 2017, 2018 and 2019, respectively. Wild *H.halys* egg masses were identified through 30-min bouts of physical inspection of preferred host plants (Table 2). Each branch was inspected up to a height of 3 m using a step ladder. The number of wild egg masses identified in the survey was 5, 8 and 106 in 2017, 2018 and 2019, respectively. Wild egg masses were collected at the time of detection.

Upon collection, all egg masses were inspected for the presence of guarding parasitoid wasps. If present, wasps were collected with an aspirator (Carolina Scientific Supply Co. Burlington, NC) and placed into a 47 mm plastic Petri dish (Fisher Scientific Co. L.L.C. Pittsburgh, PA) with the associated egg mass to allow for further oviposition during transport to the lab in a cooler at ambient temperature, 15.5–⁠24°C.

In the lab, egg masses were stored under the same conditions as the *H.halys* colony described above. Guarding female wasps were removed upon arrival at the lab, preserved in ethanol and later pinned for identification. Collected egg masses were inspected for the number of hatched (*H.halys* emergence), parasitised (parasitoid wasp emergence), missing (number of lab-reared eggs not present after field collection), unhatched or predated eggs (e.g. chewing or sucking damage) present approximately one week after collection, following procedures established by Ogburn et al. (2016). Egg masses were observed again six weeks after collection to identify late-emerging wasps or those with partially-developed wasps within eggs (Stahl et al. 2019). Wasp species were identified using the keys to Nearctic *Trissolcus* (Talamas et al. 2015a), Nearctic *Telenomus* (Johnson 1984) and Nearctic *Anastatus* (Burks 1967).

### Statistical Methods

Parasitism (defined as the proportion of egg masses in which one or more eggs produced adult wasps) was compared amongst years and egg types (wild and lab-reared) using a generalised linear model with a quasi-binomial distribution to account for over-dispersion due to small sample sizes in some years and zero-inflation. We report means and intervals that have been inverse-linked from the logit scale of the statistical model to the original proportion scale. Computations used the *glm* function in the *stats* package and various functions in the *car* (Fox and Weisberg 2019) and *emmeans* (Lenth 2019) packages in R software (R version 3.6.1; R Core Team 2019).

### Voucher Specimens

Three voucher specimens of *Trissolcusjaponicus* from this study have been deposited in the Florida State Collection of Arthropods, Gainesville, Florida (FSCA 00090589, FSCA 00090661, FSCA 00090662). A Darwin Core Archive of the data associated with these specimens is provided in Suppl. material 1.

## Results

**Figure 2.**
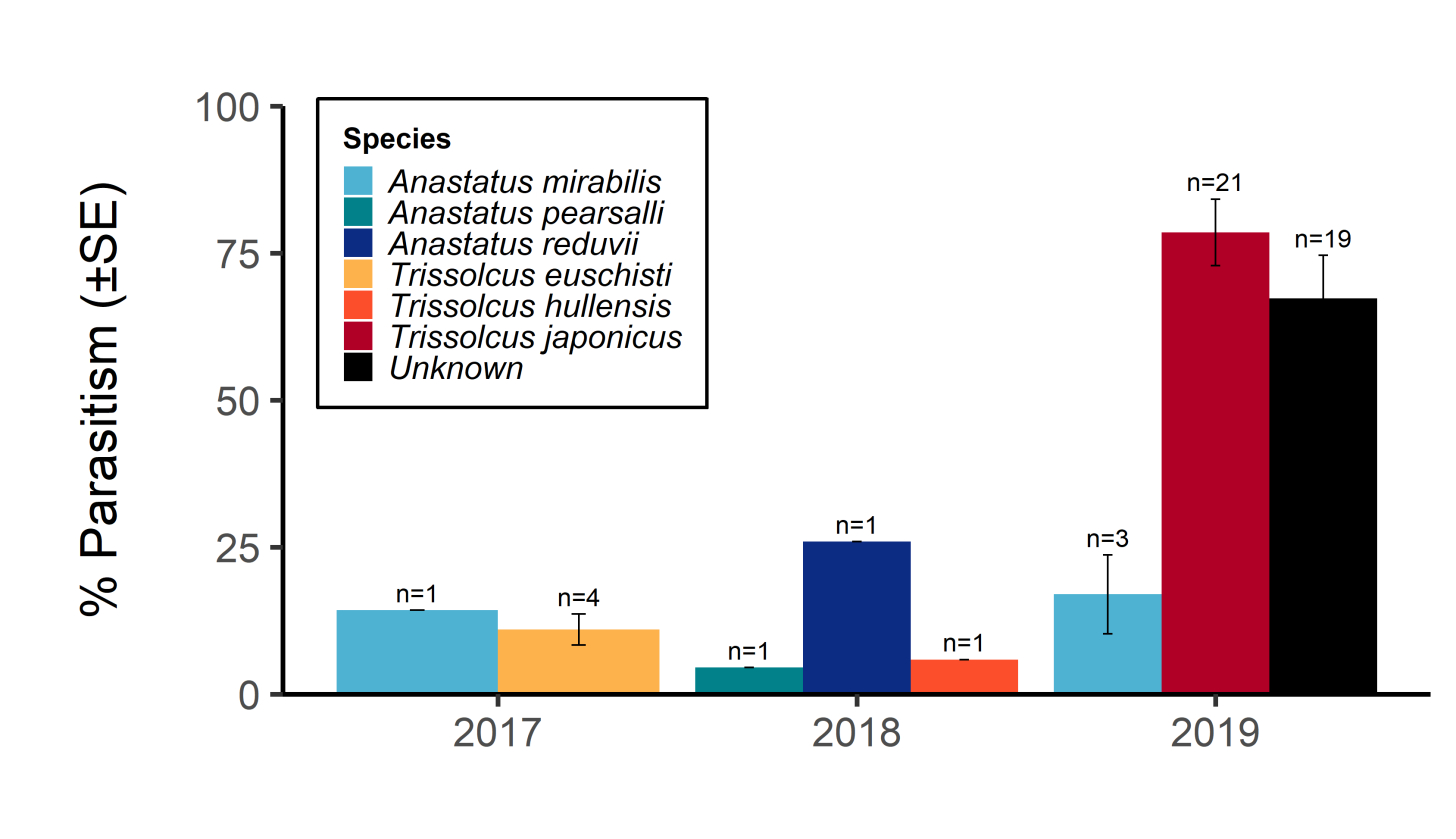
Percent parasitism (± SE) of eggs in wild and lab-reared egg masses with adult wasp emergence in northern Utah, 2017–⁠2019. Sample size (n) represents the number of egg masses parasitised by the indicated wasp species in each year. Bars without standard error lines represent single egg masses. The *Unknown* category represents egg masses in which parasitoid wasp emergence was confirmed, but no wasp specimens remained to confirm species identification.

**Figure 3.**
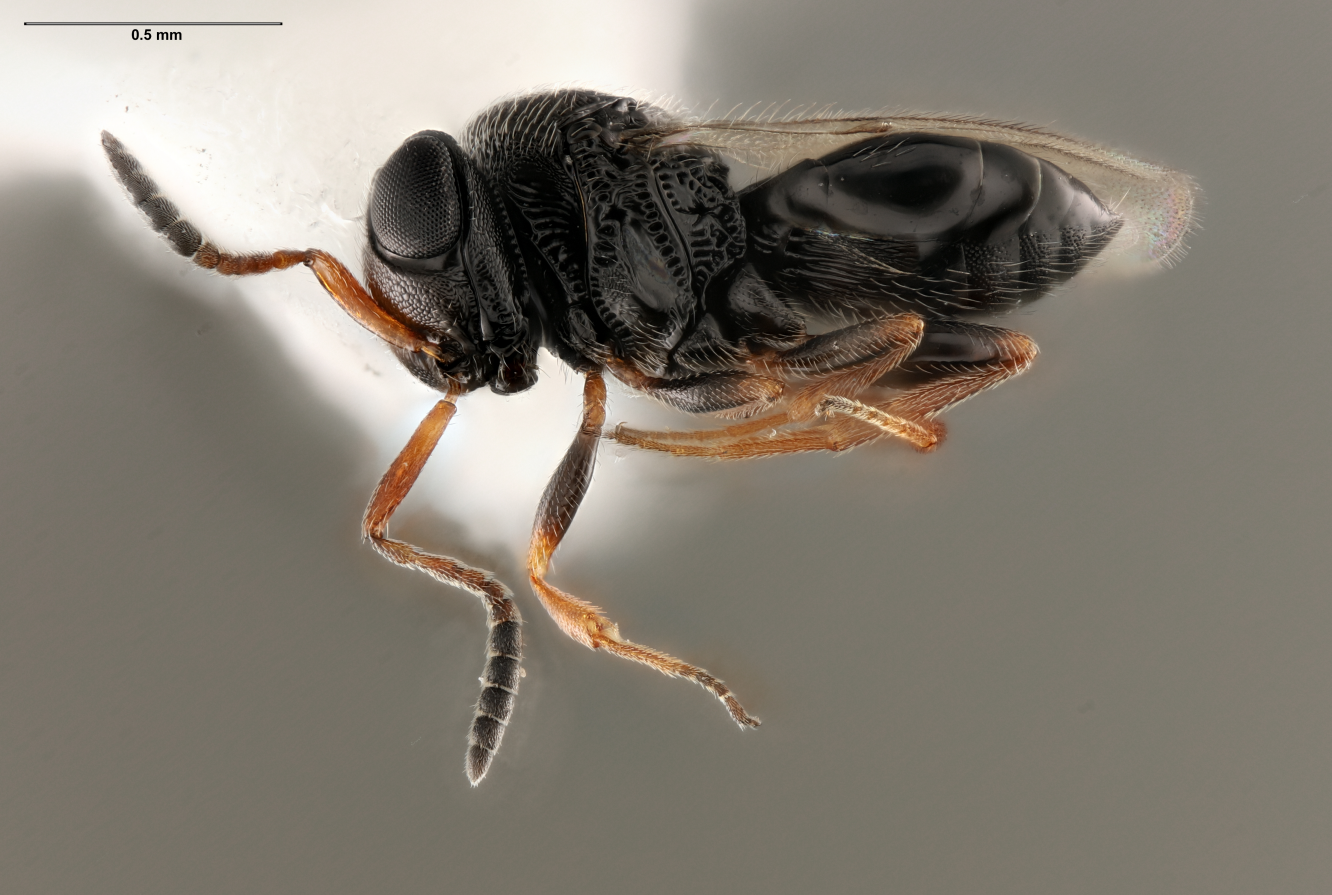
Photo of female *Trissolcusjaponicus* (FSCA 00090662), found in Salt Lake City, Site 1, on 17 June 2019. Key identifying characters include the episternal foveae occurring in a continuous line from the postacetabular sulcus to the mesopleural pit.

Over the three year survey period, a total of 39 parasitoids from five native wasp species emerged from six wild and five lab-reared *H.halys* egg masses. *Anastatusmirabilis* (Walsh & Riley), *A.pearsalli* Ashmead, *A.reduvii* Ashmead, *Trissolcuseuschisti* (Ashmead) and *T.hullensis* (Harrington) were documented from both guarding females and successful emergence from *H.halys* egg masses (Fig. 2). *Trissolcusutahensis* (Ashmead) and *Telenomuspodisi* Ashmead were observed guarding *H.halys* eggs, but did not successfully emerge as adults. *Catalpaspeciosa* (Warder), *Malusdomestica* Borkh and *Prunuspersica* (L.) Batsch were the only plant species on which lab-reared egg masses were parasitised and this parasitism was by native wasp species exclusively (Table 1). In June 2019, the Asian parasitoid *T.japonicus* was first discovered in Utah emerging from two wild *H.halys* egg masses at Site 1 in Salt Lake City (Figs 1, 2, 3) and was detected consistently from June through September. Further, a single egg mass with emergent *T.japonicus* was found at Site 17 in August 2019. *Trissolcusjaponicus* was only detected at these two suburban landscape sites. A total of 452 *T.japonicus* emerged from 21 of the 106 wild egg masses found in 2019 (Fig. 2). Parasitised wild egg masses were collected on two tree species, *C.speciosa* and *Acergrandidentatum* Nutt, with attack by *T.japonicus* occurring only on *C.speciosa* (Table 2).

When native wasp species successfully emerged from *H.halys* eggs, the mean number of parasitised eggs per affected egg mass was low, 4–25%. When considering only those egg masses giving rise to adult *T.japonicus* in 2019, the mean egg parasitism rate per mass was 78.5%. Additionally in 2019, a group of 19 wild egg masses experienced a similarly high mean parasitism rate of 67.3%, though these egg masses did not have adult wasps present at the time of collection, only signs of chewing and emergence (Fig. 2).

Mean parasitism of lab-reared egg masses was 0.42% and 0.05% in 2017 and 2018, with no wasps emerging in 2019. Mean parasitism rates of wild-collected egg masses in 2017, 2018 and 2019 were 2.9%, 3.7% and 28.2%, respectively (Table 3). The generalised linear model did not reveal a significant two-way interaction between year and egg type (P = 0.196, F_2,348_ = 1.63). Significantly more wasps emerged from wild than lab-reared egg masses (P = 0.002, F_1,348_ = 9.50). There were no significant differences in mean wasp emergence amongst years (P > 0.797, F_2,348_ = 0.23).

## Discussion

Surveys of wild and lab-reared *H.halys* eggs in northern Utah demonstrated relatively high diversity of native parasitoid wasps, but these native species all exhibited low rates of parasitism. These findings are congruent with other North American surveys of *H.halys* egg parasitoids (Dieckhoff et al. 2017, Abram et al. 2019b). Low native parasitism rates could be caused by deterrence from natural chemical defences on and within *H.halys* eggs or a lack of effective venom at the time of female oviposition needed for successful wasp development in the exotic host egg (Haye et al. 2015a, Tognon et al. 2016). Other research suggests that the use of parasitism emergence as a metric of parasitoid effectiveness underestimates native wasp effects on *H.halys* eggs since partially-developed wasps that do not complete emergence often kill the stink bug host (Abram et al. 2019a, Cornelius et al. 2016b, Schumm 2020). Although our egg dissections revealed many unhatched *H.halys* eggs with undifferentiated contents (Milnes and Beers 2019), the ultimate cause of egg death could not be ascertained.

Our results support those of Jones et al. (2014) and Abram et al. (2017) who found that wild (*in situ*) egg masses more accurately detect the presence and ability of parasitoid wasps to emerge from *H.halys* eggs than do field-deployed lab-reared egg masses. This difference may be due to a variety of factors, including the age of the egg mass upon deployment, length of egg mass exposure to field conditions and deployment height of egg masses in host trees. Hedstrom et al. (2017) noted the importance of semiochemical cues associated with the success of *T.japonicus* in finding and stinging *H.halys* egg masses. Research by Boyle et al. (2019) has also shown that kairomones, left by *H.halys* on host plant leaves, are detectable by *T.japonicus* and the wasp resides on these leaves longer than those lacking such kairomones. Therefore, lower parasitism rates of lab-reared egg masses could be due to reduced chemical cues.

Although current parasitism by the exotic *T.japonicus* in northern Utah is modest, relative to those in its native range (Yang et al. 2009, Zhang et al. 2017), our results indicate that *T.japonicus* has the potential to provide biological control of *H.halys* in the Intermountain West. Parasitism rates were not shown to be different amongst years, but our data clearly show higher parasitism in 2019, when *T.japonicus* was discovered, as compared to previous years. The dissonance of biological and statistical conclusions in our results is likely due to the variable and low sample size of egg masses. *Trissolcusjaponicus* may have killed more *H.halys* eggs than we were able to document, based on identification of the causal wasp. Indeed, many egg masses were attacked by parasitoids that had already emerged from eggs before collection in 2019, with higher mean parasitism rates in affected egg masses than those observed for native wasp species, suggesting that at least some of the unidentified parasitoids were *T.japonicus*. It is also of interest to point out that *T.japonicus* was detected in two suburban landscape sites in Salt Lake County in 2019 and not in agricultural sites.

The northern Utah region differs in its climate and topography from most locations in which *T.japonicus* has been documented or predicted to become established in North America (Avila and Charles 2018). Given the arid, high elevation conditions of northern Utah that include cold winters and hot summers, detection of an adventive *T.japonicus* population implies potential for range expansion into other locations within the greater Intermountain West region. These results support the possibility of an eventual intersection of eastern and western *T.japonicus* populations in North America (Jarrett et al. 2019, Talamas et al. 2015b, Milnes et al. 2016). Further research should focus on the capacity of *T.japonicus* to persist in the Intermountain West, specifically focusing on overwintering behaviour where heavy snowfall accumulation and consistent sub-zero temperatures occur (Lowenstein et al. 2019, Nystrom Santacruz et al. 2017). In fact, follow-up surveys in 2020 have documented continued detection of *T.japonicus* in Salt Lake and expansion into Utah counties (K. Richardson, personal communication). Laboratory rearing and releases, in conjunction with conservation efforts, are critical next steps in supporting the future establishment of *T.japonicus* populations in Utah.

## Conclusions

Our findings show that an adventive population of *T.japonicus* in northern Utah is causing higher levels of reproductive parasitism of *H.halys* eggs compared to native wasp species and wild (*in situ*) egg masses provide a more accurate measure of parasitoid activity compared to those deployed from lab colonies. This study reports the first detection of *T.japonicus* in the Intermountain West, a novel geographic location for this parasitoid in North America.

## Supplementary Material

FF212277-A5F9-5D12-B1EE-1431A9556DCC 10.3897/BDJ.8.e53363.suppl1 Supplementary material 1 Darwin Core Archive of *Trissolcusjaponicus* voucher specimens Data type Occurrences Brief description Three female specimes of *Trissolcusjaponicus* are deposited in the Florida State Collection of Arthropods. The attached file provides their occurrence data in Darwin Core format. File: oo_423049.xls https://binary.pensoft.net/file/423049 Elijah Talamas

136D0940-76DE-5317-B218-81656C73F37B 10.3897/BDJ.8.e53363.suppl2 Supplementary material 2 Comprehensive data file for all lab-reared (H) and wild (N) egg mass parasitism in Utah 2017–2019 Data type Counts and Occurrences Brief description Archive of all lab-reared (H) and wild collected (N) *Halyomorphahalys* egg masses inspected in northern Utah 2017–2019. File: oo_429562.csv https://binary.pensoft.net/file/429562 Mark Cody Holthouse and Zachary R. Schumm

AD376E0C-F9B4-5D32-8CA9-DED12064F0C0 10.3897/BDJ.8.e53363.suppl3 Supplementary material 3 Jupypter Notebook of GLM Model Data type Jupyter Notebook (R code) Brief description This file can be opened on Jupyter Notebook. The file contains R code displaying the generalised linear model used to compare mean parasitism by egg type and year in northern Utah. File: oo_429561.html https://binary.pensoft.net/file/429561 Mark Cody Holthouse

## Figures and Tables

**Table 1.** Number of deployed and parasitised fresh lab-reared *H.halys* egg masses by native wasps on multiple plant species in northern Utah from June through September, 2017 –⁠ 2019. Parasitism denotes adult wasp emergence.

**Plant Species**	**Total Egg Masses (Eggs)**	**Parasitised Egg Masses (Eggs)**
*Acernegundo*	13 (311)	0 (0)
*Ailanthusaltissima*	7 (187)	0 (0)
*Catalpaspeciosa*	62 (1503)	1 (5)
*Cerciscanadensis*	19 (474)	0 (0)
*Elaeagnusangustifolia*	5 (137)	0 (0)
*Helianthusannuus*	1 (25)	0 (0)
*Malusdomestica*	48 (1099)	1 (1)
*Malus* sp.	6 (122)	0 (0)
*Prunusarmeniaca*	11 (277)	0 (0)
*Prunuscerasus*	12 (298)	0 (0)
*Prunusdomestica*	3 (83)	0 (0)
*Prunuspersica*	17 (453)	3 (7)
*Robiniapseudoacacia*	3 (73)	0 (0)
*Sambucus* sp.	6 (168)	0 (0)
*Zeamays*	22 (559)	0 (0)

**Table 2.** Number of deployed and parasitised wild *H.halys* egg masses by all wasps, native and exotic, on multiple plant species in northern Utah from June through September, 2017–⁠2019. Parasitism denotes adult wasp emergence.

**Plant Species**	**Year**	**Total Egg Masses (Eggs)**	**All Parasitised Egg Masses (Eggs)**	Egg Masses Parasitized by *T.japonicus* (Eggs)*
*Acergrandidentatum*	2018	1 (22)	1 (1)	0 (0)
*Catalpaspeciosa*	2017	4 (108)	1 (4)	0 (0)
	2018	6 (164)	1 (7)	0 (0)
	2019	105 (2791)	43 (796)	21 (452)
*Prunuscerasus*	2018	1 (28)	0 (0)	0 (0)
	2019	1 (28)	0 (0)	0 (0)
*Zeamays*	2017	1 (28)	0 (0)	0 (0)

* Wasp identity confirmed upon adult emergence from *H.halys* eggs.

**Table 3.** Table 3. Mean parasitism of lab-reared and wild egg masses collected in northern Utah, 2017–⁠2019. LCL and UCL refer to the lower and upper limit of a 68% confidence interval, respectively and approximately depict the mean +/- 1 SE. See Suppl. materials 2, 3.

**Egg Type**	**Year**	**Mean Parasitism (%)**	**LCL**	**UCL**
Lab-reared	2017	0.42	0.19	0.94
Wild	2017	2.94	0.74	11.00
Lab-reared	2018	0.05	0.00	0.73
Wild	2018	3.74	1.41	9.53
Lab-reared	2019	0.00	0.00	100.00
Wild	2019	28.20	25.90	30.64
