# Supplementary material for: Surveys in northern Utah for egg parasitoids of Halyomorphahalys (Stål) (Hemiptera: Pentatomidae) detect Trissolcusjaponicus (Ashmead) (Hymenoptera: Scelionidae)
Source: Biodivers Data J. 2020 Aug 13;8:e53363. doi: 10.3897/BDJ.8.e53363 (PMC7441073; doi:10.3897/BDJ.8.e53363)
Supplement: Supplementary material 3 — Jupypter Notebook of GLM Model [file bdj-08-e53363-s003.html]

UT\_Parasitioid\_Code


In [ ]:

```
#Set the working directory
setwd('C:/Users/mchol/Desktop/BMSB Research/2019/Wasp Data')

##### GLM code for comparing parasitism means ######
library(emmeans)
library(car)
## Loading required package: carData
Data2=read.csv(file="Parasitism_Data_BDJ.csv", header=TRUE, sep=",")
Data2$Year= as.factor(Data2$Year)

options(contrasts = c("contr.sum", "contr.poly"))

#fit the model with quasibinomial family to account for overdispersion
TYpar.glm = glm(cbind(Par, Total_Eggs-Par) ~ EggType*Year, family = quasibinomial(), data = Data2)
summary(TYpar.glm)
## 
## Call:
## glm(formula = cbind(Par, Total_Eggs - Par) ~ EggType * Year, 
##     family = quasibinomial(), data = Data2)
## 
## Deviance Residuals: 
##     Min       1Q   Median       3Q      Max  
## -4.3868  -0.4875  -0.3880  -0.1328   8.4151  
## 
## Coefficients:
##                Estimate Std. Error t value Pr(>|t|)
## (Intercept)      -6.642    139.118  -0.048    0.962
## EggType1         -4.083    139.118  -0.029    0.977
## Year1             2.164    139.118   0.016    0.988
## Year2             1.174    139.120   0.008    0.993
## EggType1:Year1    3.101    139.118   0.022    0.982
## EggType1:Year2    1.863    139.120   0.013    0.989
## 
## (Dispersion parameter for quasibinomial family taken to be 7.753359)
## 
##     Null deviance: 4517.3  on 353  degrees of freedom
## Residual deviance: 2665.2  on 348  degrees of freedom
## AIC: NA
## 
## Number of Fisher Scoring iterations: 15
Anova(TYpar.glm, type=3, test.statistic=c("F"))
## Analysis of Deviance Table (Type III tests)
## 
## Response: cbind(Par, Total_Eggs - Par)
## Error estimate based on Pearson residuals 
## 
##               Sum Sq  Df F values   Pr(>F)   
## EggType        73.64   1   9.4984 0.002221 **
## Year            3.52   2   0.2267 0.797256   
## EggType:Year   25.35   2   1.6349 0.196457   
## Residuals    2698.17 348                     
## ---
## Signif. codes:  0 '***' 0.001 '**' 0.01 '*' 0.05 '.' 0.1 ' ' 1
#Model code produces our means and deviance from the means by way of LCL and UCL
# Level has been set to 0.68 to act as an equivalent to (+/-) 1 SE
#This output is reported in Table 3 which compares wild (N) vs. lab-reared (H) egg mass parasitism
emmeans(TYpar.glm, ~ EggType + Year, type = "response", level=0.68)  
##  EggType Year     prob       SE  df asymp.LCL asymp.UCL
##  H       2017 4.23e-03 3.40e-03 Inf  1.91e-03   0.00938
##  N       2017 2.94e-02 4.03e-02 Inf  7.38e-03   0.10996
##  H       2018 4.58e-04 1.28e-03 Inf  2.87e-05   0.00726
##  N       2018 3.74e-02 3.61e-02 Inf  1.41e-02   0.09530
##  H       2019 1.00e-08 4.54e-06 Inf  0.00e+00   1.00000
##  N       2019 2.82e-01 2.36e-02 Inf  2.59e-01   0.30643
## 
## Confidence level used: 0.68 
## Intervals are back-transformed from the logit scale
sessionInfo()
## R version 3.6.3 (2020-02-29)
## Platform: x86_64-w64-mingw32/x64 (64-bit)
## Running under: Windows 10 x64 (build 18363)
## 
## Matrix products: default
## 
## locale:
## [1] LC_COLLATE=English_United States.1252 
## [2] LC_CTYPE=English_United States.1252   
## [3] LC_MONETARY=English_United States.1252
## [4] LC_NUMERIC=C                          
## [5] LC_TIME=English_United States.1252    
## 
## attached base packages:
## [1] stats     graphics  grDevices utils     datasets  methods   base     
## 
## other attached packages:
## [1] car_3.0-7     carData_3.0-3 emmeans_1.4.6
## 
## loaded via a namespace (and not attached):
##  [1] zip_2.0.4         Rcpp_1.0.4.6      plyr_1.8.6        cellranger_1.1.0 
##  [5] pillar_1.4.3      compiler_3.6.3    highr_0.8         forcats_0.5.0    
##  [9] tools_3.6.3       digest_0.6.25     lifecycle_0.2.0   tibble_3.0.1     
## [13] evaluate_0.14     lattice_0.20-38   pkgconfig_2.0.3   rlang_0.4.5      
## [17] openxlsx_4.1.4    Matrix_1.2-18     curl_4.3          mvtnorm_1.1-0    
## [21] haven_2.2.0       xfun_0.13         rio_0.5.16        coda_0.19-3      
## [25] stringr_1.4.0     knitr_1.28        vctrs_0.2.4       hms_0.5.3        
## [29] grid_3.6.3        data.table_1.12.8 readxl_1.3.1      survival_3.2-3   
## [33] foreign_0.8-75    rmarkdown_2.1     multcomp_1.4-13   TH.data_1.0-10   
## [37] magrittr_1.5      ellipsis_0.3.0    codetools_0.2-16  htmltools_0.4.0  
## [41] splines_3.6.3     MASS_7.3-51.5     abind_1.4-5       xtable_1.8-4     
## [45] sandwich_2.5-1    stringi_1.4.6     estimability_1.3  crayon_1.3.4     
## [49] zoo_1.8-7
```
